# RETRACTED: Tarawneh et al. Berberine Inhibited Growth and Migration of Human Colon Cancer Cell Lines by Increasing Phosphatase and Tensin and Inhibiting Aquaporins 1, 3 and 5 Expressions. *Molecules* 2023, *28*, 3823

**DOI:** 10.3390/molecules31010112

**Published:** 2025-12-29

**Authors:** Noor Tarawneh, Lama Hamadneh, Bashaer Abu-Irmaileh, Ziad Shraideh, Yasser Bustanji, Shtaywy Abdalla

**Affiliations:** 1 Department of Biological Sciences, School of Science, The University of Jordan, Amman 11942, Jordan; 2 Department of Pharmacy, Faculty of Pharmacy, Al-Zaytoonah University, Amman 11733, Jordan; 3 Department of Basic Medical Sciences, Al-Balqa Applied University, Al-Salt 19117, Jordan; 4 Hamdi Mango Center for Scientific Research, The University of Jordan, Amman 11942, Jordan; 5 Department of Biopharmaceutics and Clinical Pharmacy, School of Pharmacy, The University of Jordan, Amman 11942, Jordan; 6 Department of Basic Medical Sciences, College of Medicine, University of Sharjah, Sharjah 27272, United Arab Emirates

The journal retracts the article “Berberine Inhibited Growth and Migration of Human Colon Cancer Cell Lines by Increasing Phosphatase and Tensin and Inhibiting Aquaporins 1, 3 and 5 Expressions” [1] cited above.

Following publication, concerns were brought to the attention of the Editorial Office regarding the integrity of an image presented in this publication [1].

Adhering to our standard procedure, an investigation was conducted by the Editorial Office and Editorial Board that identified indications of inappropriate editing and partial duplication within Figure 5 of this article. Although the authors cooperated with the investigation and submitted processed material, full raw material meeting the journals requirements for original images could not be provided for Editorial Board evaluation.

Consequently, the Editorial Board has lost confidence in the reliability of the findings and has decided to retract this publication, as per MDPI’s retraction policy (https://www.mdpi.com/ethics#_bookmark30).

This retraction was approved by the Editor-in-Chief of the *Molecules* journal.

The authors did not agree to this retraction.
